# Classification Prediction of Breast Cancer Based on Machine Learning

**DOI:** 10.1155/2023/6530719

**Published:** 2023-01-11

**Authors:** Hua Chen, Nan Wang, Xueping Du, Kehui Mei, Yuan Zhou, Guangxing Cai

**Affiliations:** School of Science, Hubei University of Technology, Wuhan 430000, China

## Abstract

Breast cancer is the most common and deadly type of cancer in the world. Based on machine learning algorithms such as XGBoost, random forest, logistic regression, and K-nearest neighbor, this paper establishes different models to classify and predict breast cancer, so as to provide a reference for the early diagnosis of breast cancer. Recall indicates the probability of detecting malignant cancer cells in medical diagnosis, which is of great significance for the classification of breast cancer, so this article takes recall as the primary evaluation index and considers the precision, accuracy, and *F*1-score evaluation indicators to evaluate and compare the prediction effect of each model. In order to eliminate the influence of different dimensional concepts on the effect of the model, the data are standardized. In order to find the optimal subset and improve the accuracy of the model, 15 features were screened out as input to the model through the Pearson correlation test. The K-nearest neighbor model uses the cross-validation method to select the optimal *k* value by using recall as an evaluation index. For the problem of positive and negative sample imbalance, the hierarchical sampling method is used to extract the training set and test set proportionally according to different categories. The experimental results show that under different dataset division (8 : 2 and 7 : 3), the prediction effect of the same model will have different changes. Comparative analysis shows that the XGBoost model established in this paper (which divides the training set and test set by 8 : 2) has better effects, and its recall, precision, accuracy, and *F*1-score are 1.00, 0.960, 0.974, and 0.980, respectively.

## 1. Introduction

In the past ten years, the incidence of breast cancer in China has increased by 47%, and the incidence is increasing year by year, and the incidence of breast cancer is gradually younger [1]. The pathogenesis of breast cancer is related to personal hormones, family history, marriage, and childbearing history [2]. Breast cancer is not easy to detect in the early stage, and has the characteristics of the early age of onset but late presentation [3, 4]. At present, the main diagnosis of breast cancer is based on three methods: puncture cytology [5], ultrasound scan [6], and mammogram X-ray [7]. If a patient is caught early in breast cancer, the more likely it is to be cured and the better the prognosis. Therefore, regular examination and early diagnosis are very necessary for the prevention and timely detection of breast cancer.

In the medical field, the establishment of models through machine learning methods can assist doctors to improve the detection rate of cancer, so as to achieve the purpose of early detection and early treatment. Machine learning methods have yielded good results in the diagnosis of cancer [8, 9]. Wu et al. [10] observed the cell morphology under the microscope and found that there were obvious differences between breast cancer cells and normal healthy cell parameters. This finding provides a theoretical basis for many studies. While there are many machine learning methods currently applied to breast cancer cell classification, no single algorithm can be applied to all problems. Each type of machine learning algorithm has its own areas of expertise, so the choice of algorithm is different in different scenarios.

Shen et al. [11] used the XGBoost model to classify and predict breast cancer, and the accuracy reached 97.86%, and the recall reached 95.83%. Deng et al. [12] used the XGBoost algorithm to classify and predict breast cancer with an accuracy of 0.96 and a recall of 0.97. Monirujjaman Khan et al. [13] used multiple machine learning models to identify breast cancer, and random forest, decision tree, K-nearest neighbor, and logistic regression were the algorithms with higher *F*1-score, 96%, 95%, 90%, and 98%, respectively. Bhardwaj et al. [14] used multilayer perceptron (MLP), K-nearest neighbor (KNN), genetic algorithm (GP), and random forest (RF) to classify benign and malignant breast cancer cells, and the experimental results showed that the optimal classifier was RF with a classification accuracy of 96.24%. Dong and Ma [15] studied the possible markers of triple-negative breast cancer, and machine learning algorithms were used to predict whether people had triple-negative breast cancer. The results show that the accuracy of the support vector machine (SVM) classification prediction model reaches 97.8%. In order to improve the accuracy of breast cancer identification methods and improve machine learning algorithms, Wang et al. [16] proposed a weighted AUC ensemble learning model based on SVM for breast cancer diagnosis, using C-SVM and V-SVM with 6 kernel functions to increase the diversity of the base model set and comparing different decision results with the Area Integration (WAUCE) model under the weighted receiving working characteristic curve. The results show that on the small dataset, the proposed WAUCE structure reduces the variance of the diagnostic accuracy by up to 69.23% and improves the accuracy by 0.94%. Zheng et al. [17] tested the Wisconsin Breast Cancer (WDBC) dataset according to the K-means and support vector machine hybrid algorithm extracts tumor features and diagnoses breast cancer, and the results show that the hybrid algorithm improves the accuracy to 97.38%. Jia et al. [18] proposed a new population optimization algorithm, Whale Optimization Algorithm (WOA), which intelligently adjusts the parameters of the SVM model, and the experimental results show that the performance of the WOA-SVM model is significantly better than that of the traditional breast cancer recognition model, with an accuracy of 97.5%. In order to solve the problem of overfitting of machine learning techniques in breast cancer classification, Singh et al. [19] proposed a functionally connected artificial neural network (FLANN) and experimentally found that the model has high accuracy for early diagnosis of breast cancer. Mahesh et al. [20] propose a breast cancer prediction XGBoost ensemble technique based on known feature patterns, first using synthetic minority oversampling technology (SMOTE) to deal with data imbalance and noise problems and then using naïve Bayes classifier, decision tree classifier, and random forest, respectively, combined with XGBoost and classifying the data. According to experimental analysis, XGBoost-Random Forest ensemble classifier has an accuracy rate of 98.20% in the early detection of breast cancer.

Based on XGBoost, random forest, logistic regression, K-nearest neighbor, and other machine learning methods, this paper establishes different models to classify and predict breast cancer, which provides a reference for early diagnosis of breast cancer. When most studies apply machine learning models to breast cancer cell diagnosis, they focus on using the precision, accuracy, and *F*1-score of the model as indicators to evaluate the quality of the model, while ignoring the medical diagnostic significance of the recall of the model, which indicates the proportion of malignant breast cancer cells that are predicted, and the higher the recall, the greater the probability of malignant cells being predicted in breast cancer cells. Therefore, this article takes recall as the primary index and considers precision, accuracy, and *F*1-score to evaluate the model used.

In the modeling process, data preprocessing is a very important part, and the effect of the predictive model is different depending on the processing method. In order to eliminate the influence of different dimensional concepts on the effect of the model, the data is standardized. In order to find the optimal subset and improve the accuracy of the model, feature selection was made according to the Pearson correlation coefficient between the feature variable and the target variable. For the problem of positive and negative sample imbalance, the hierarchical sampling method is used to extract the training set and test set proportionally according to different categories. Considering that the prediction effect of machine learning models varies under different dataset divisions, this paper will use different dataset divisions (8 : 2 and 7 : 3) as two sets of experiments to observe the prediction effect of the model established in this paper.

## 2. Data Preprocessing

### 2.1. Data Introduction

The data set used in this paper is the breast cancer data in the UCI data set, which was provided by the famous Dr. William from the Clinical Medicine Research Institute of the University of Wisconsin [21]. Features are computed from a digitized image of a fine needle aspirate (FNA) of a breast mass. They describe the characteristics of the cell nuclei present in the image. The data set contained 569 experimental samples, including 357 benign samples and 212 malignant samples of breast cancer. For the cells extracted from each experimental object, the following ten features of its nucleus are mainly collected: radius (mean of the distance from center to points on the perimeter), perimeter, smoothness (local variation in radius lengths), area, compactness (perimeter*∗∗*2/area-1.0), concavity (severity of concave portions of the contour), symmetry, texture (standard deviation of gray-scale values), concave points (number of concave portions of the contour), and fractal_dimension (“coastline approximation”-1). The mean, standard error, and “worst” or largest (mean of the three largest values) of these features were computed for each image, resulting in 30 features. The classification label represents the type of breast cancer. Therefore, the sample data set contains a total of 30 features and one sample label feature (malignant and benign).

### 2.2. Data Standardization

By observing the value range of each feature, it is found that the data values of different features differ greatly. In some models, different dimensions have a great influence on the prediction effect. For example, the k-nearest neighbor algorithm based on distance division needs to keep the data dimension consistent, so the data need to be standardized before modeling. However, some models are less affected by dimensionality, such as the random forest algorithm. In order to make the experiment comparative, the data of different models are treated in the same way.

For problems with different sample data dimensions, the commonly used dimensionless processing methods include data standardization, and data standardization methods include Min-max standardization and *Z*-score standardization. Among them, when the data used have outliers outside the value range, or the maximum and minimum values of some indicators are unknown, the *Z*-score standardization can be used.

In this paper, according to the characteristics of the WDBC breast cancer dataset, the *Z*-score standardization was selected to process the data. The data processed by the *Z*-score standardization [22] follows a standard normal distribution, that is, the mean is 0 and the variance is 1. The formula for *Z*-score standardization is as follows:

(1) $$\begin{array}{c} x^{*}=\frac{x-mean}{std}\text{,} \end{array}$$

where mean is the mean of sample characteristic data and std is the standard deviation of sample characteristic data. The data standardization results are shown in Table 1.

### 2.3. Feature Selection

As an important part of the data preprocessing process, feature selection is to find the optimal subset, feature selection can reduce redundant and useless features to improve the accuracy of the model. The feature selection method is generally divided into the overthinking method, the encapsulation method, and the embedding method. The filtering method can be independent of the algorithm used later in the study and has high computational efficiency and strong generalization ability [23], so the feature selection method in this paper uses the filter method, and the general method summary in the filtering method is shown in Table 2.

The breast cancer data after 0 mean normalization meet the requirements of the Pearson correlation coefficient test in the filtering method; so in this paper, Pearson's correlation coefficient [24] is used to test the correlation between each feature and the target variable. Pearson's correlation coefficient formula is as follows:

(2) $$\begin{array}{c} \rho_{X_{1}X_{2}}=\frac{Cov\left(X_{1},X_{2}\right)}{\sqrt{{DX}_{1}*{DX}_{2}}}=\frac{{EX}_{1}X_{2}-{EX}_{1}*{EX}_{2}}{\sqrt{{DX}_{1}*{DX}_{2}}}\text{,} \end{array}$$

where *ρ*_*X*_1_*X*_2__ represents the correlation coefficient between two variables, Cov(*X*_1_, *X*_2_) represents the covariance between two variables, EX_1_ represents the expectation of variables, and DX_1_ represents the variance of variables.

According to the Pearson correlation coefficient, there are 15 features whose absolute value of the correlation coefficient with the target variable is greater than or equal to 0.5. These 15 feature variables are used for model construction, and the 15 feature and target variables are shown in Table 3.

## 3. Model Construction

In this paper, the categories of breast cancer are predicted based on XGBoost, random forest, logistic regression, and K-nearest Neighbor model, respectively. Malignant breast cancer is regarded as a positive sample, while benign breast cancer is regarded as a negative sample.

To solve the problem of sample imbalance, this paper uses a stratified sampling method [25] to extract the training set and test set in proportion to all kinds of sample data. Stratified sampling is also called type sampling. The sample population is divided into subpopulations that are independent of each other. Random sampling was carried out in proportion in each subpopulation. Stratified sampling draws a more representative sample and is more suitable for unbalanced samples.

Different data set partitioning may lead to different model effects. Therefore, this paper carries out two groups of experiments according to a different division of the sample data set. The first group divided the data set into a training set and test set in a ratio of 8 : 2, and the second group divided the data set into a training set and test set in a ratio of 7 : 3. Observe the model performance of the four algorithms under different data set partitioning.

### 3.1. Evaluation Indicators

In this study, accuracy, precision, recall, and *F*1-score [26, 27] were used to evaluate the prediction effect of the model. Considering the particularity of medical diagnosis, it is expected that all malignant breast cancer can be predicted. Therefore, recall is taken as an important evaluation index here. The higher the recall is, the higher the proportion of malignant breast cancer that can be predicted. The model classification results can generate a confusion matrix [28], as shown in Table 4.

Here, TP is a true positive, indicating the number of positive samples predicted as positive samples. TN is a true negative, indicating the number of negative samples predicted as negative samples. FP is a false positive, indicating the number of positive samples predicted from negative samples, which is called type 1 error. FN is a false negative, indicating the number of positive samples predicted as negative samples, which is called type 2 error.

Precision, abbreviated as *P*. For the predicted results, precision represents how many of the positive predicted samples are really positive samples, and the formula is

(3) $$\begin{array}{c} Precision=\frac{TP}{TP+FP}\text{.} \end{array}$$

Recall is also known as the true positive rate. For the original samples, the recall represents how many positive samples in the samples are predicted correctly, and the formula is

(4) $$\begin{array}{c} Recall=\frac{TP}{TP+FN}\text{.} \end{array}$$

Accuracy, referred to as *A*, refers to the proportion of all correctly predicted samples (including positive samples and negative samples) in the total sample. The formula is

(5) $$\begin{array}{c} Accuracy=\frac{TP+TN}{TP+TN+FP+FN}\text{.} \end{array}$$

*F*1-score is obtained by the weighted harmonic average of precision and recall due to the contradiction between the two evaluation indexes. *F*1-score is a comprehensive evaluation index of external methods, and a higher value indicates that the classification results are more effective. The formula of index *F*1-score is

(6) $$\begin{array}{c} F1=\frac{2\text{PR}}{\left(P+R\right)}\text{.} \end{array}$$

### 3.2. Prediction Model of Breast Cancer Based on XGBoost

XGBoost, short for extreme gradient boosting, is a Boosting algorithm [29]. Both XGBoost and random forest are integration algorithms based on the decision tree. Different from the Bagging algorithm, Boosting algorithm builds weak learners one by one, accumulating multiple weak learners through continuous iteration [30]. The objective function is

(7) $$\begin{array}{c} obj=\overset{m}{\underset{i=1}{\sum }}l\left(y_{i},{\hat{y}}_{i}\right)+\overset{K}{\underset{k=1}{\sum }}\Omega \left(f_{k}\right)\text{,} \end{array}$$

where *i* represents the *i*th sample, *m* represents the sample size corresponding to the *k*th decision tree, and *K* represents the currently established weak learner. The first part of the objective function is the loss function, which measures the difference between the predicted value and the true value. The second part of the function represents the complexity of the model.

In order to optimize the tree after the *t*-th iteration, Taylor expansion is performed on the objective function. Then, the objective function can be converted into

(8) $$\begin{array}{c} obj=\overset{m}{\underset{i=1}{\sum }}\left[f_{t}\left(x_{i}\right)g_{i}+\frac{1}{2}{\left(f_{t}\left(x_{i}\right)\right)}^{2}h_{i}\right]+\Omega \left(f_{t}\right)\text{,} \end{array}$$

where *g*_*i*_ is the first derivative of loss function $l\left(y_{i}^{t},{\hat{y}}_{i}^{\left(t-1\right)}\right)$ with respect to ${\hat{y}}_{i}^{\left(t-1\right)}$, and *h*_*i*_ is the second derivative of loss function $l\left(y_{i}^{t},{\hat{y}}_{i}^{\left(t-1\right)}\right)$ with respect to ${\hat{y}}_{i}^{\left(t-1\right)}$. The regularization expression of *L*2 in the formula is

(9) $$\begin{array}{c} \Omega \left(f_{t}\right)=\gamma^{T}+\frac{1}{2}\lambda {\left\|\omega \right\|}^{2}\text{.} \end{array}$$

The addition of regular term can reduce the variance of the model and make the model obtained by the training set more simple, so as to prevent the occurrence of overfitting.

The XGBoost algorithm is used to train the model on the training data set. In the process of model training, the parameters are adjusted to obtain a better set of parameters, and finally the optimal prediction model is obtained. The model was used to predict breast cancer categories on the test set.

When the data set was divided into a training set and test set by 8 : 2, the accuracy, precision, recall, and *F*1-score of the XGBoost model were 0.974, 0.960, 1.00, and 0.980, respectively. When the XGBoost model was divided into a training set and test set by 7 : 3, the accuracy, precision, recall, and *F*1-score of the XGBoost model were 0.959, 0.946, 0.991, and 0.968, respectively. The results show that the XGBoost model has better prediction performance when the data set is divided by 8 : 2. The recall rate of 1 indicates that the XGBoost model correctly predicted all malignant breast cancers in the sample, which is very important for medical diagnosis.

### 3.3. Prediction Model of Breast Cancer Based on Random Forest

Random forest is a supervised learning algorithm that integrates multiple trees through the Bagging idea [31–33]. The bootstrap method is used to extract the training sample set from the original sample data, and the corresponding decision tree model is trained for each training set. Finally, all base classifiers are voted on, and the one with the most votes is the final category.

When the data set was divided into a training set and test set by 8 : 2, the accuracy, precision, recall, and *F*1-score of the random forest model were 0.965, 0.947, 1.00, and 0.973, respectively. When the data set was divided into a training set and test set by 7 : 3, accuracy, precision, recall, and *F*1-score were 0.953, 0.946, 0.981, and 0.963, respectively. The results show that the random forest model has better prediction performance when the data set is divided by 8 : 2. The recall rate of this model was also 1, indicating that the random forest model also correctly predicted all malignant breast cancer.

### 3.4. Prediction Model of Breast Cancer Based on Logistic Regression

LR, Logistic Regression, is one of the most widely used methods in medical data analysis [34, 35]. Logistic regression is a sigmoid function nested on the basis of a multiple linear regression model. The basic form is

(10) $$\begin{array}{c} y\left(x\right)=\frac{\exp\;\left(\theta_{0}+\theta_{1}x_{1}+\ldots +\theta_{k}x_{k}\right)}{1+\exp\;\left(\theta_{0}+\theta_{1}x_{1}+\ldots +\theta_{k}x_{k}\right)}\text{,} \end{array}$$

where *θ*_0_, *θ*_1_ … *θ*_*k*_ is similar to the regression coefficient in multiple linear regression.

When the data set was divided into a training set and test set by 8 : 2, accuracy, precision, recall, and *F*1-score of the logistic regression model were 0.947, 0.923, 1.00, and 0.960, respectively. When the data set was divided into a training set and test set by 7 : 3, accuracy, precision, recall, and *F*1-score were 0.947, 0.922, 1.00, and 0.960, respectively. It can be seen from the results that the prediction effect of the logistic regression model is consistent under the two partitioning conditions. The recall was also 1, indicating that all malignant breast cancer was correctly predicted by the logistic regression model.

### 3.5. Prediction Model of Breast Cancer Based on K-Nearest Neighbor

The K-nearest neighbor algorithm [36, 37] projects samples into higher dimensional space according to variable values. Similar samples show spatial aggregation in higher dimensional space. Euclidean distance is commonly used to measure distances in k-nearest neighbors, and the calculation method is as follows:

(11) $$\begin{array}{c} d\left(x_{i},x_{j}\right)={\left(\overset{n}{\underset{l=1}{\sum }}{\left|x_{i}^{l}-x_{j}^{l}\right|}^{2}\right)}^{1/2}\text{,} \end{array}$$

where *x*_*i*_, *x*_*j*_ represents two different samples, *x*_*i*_^*l*^ represents the value of sample *i* on attribute *l*, and *x*_*j*_^*l*^ represents the value of sample *j* on attribute *l*.

The three basic elements of the k-nearest neighbor algorithm are distance measurement, *k*-value selection, and classification decision rule.

**Figure 1:**
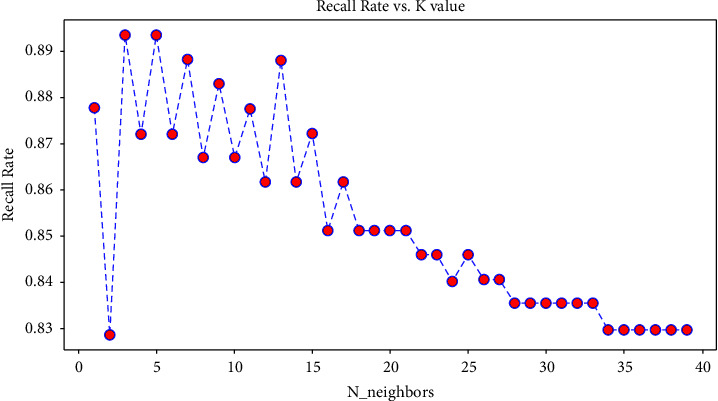
Recall rates of different *K* values.

For the problem of *K* value selection in the k-nearest neighbor algorithm, this paper uses the tenfold cross-validation method [38, 39] and takes the recall rate as the model evaluation index to select an appropriate *k* value. Let the value range of *k* be 1–40, and for each *k*, the cross-validation of tenfold is performed. The *k* value with the maximum recall rate is the optimal *k* value. The recall of different *k* values under the cross-validation of tenfold is shown in Figure 1.

It can be seen from Figure 1 that as *k* value increases, the recall decreases. When *k* = 3 and *k* = 5, the recall is the largest. Because the *k* value is set too small, it is easy to overfit, so the *k* value is set as 5 here.

When the data set was divided into a training set and test set by 8 : 2, the accuracy, precision, recall, and *F*1-score of the k-nearest Neighbor model were 0.912, 0.888, 0.986, and 0.934, respectively. When the data set was divided into a training set and test set by 7 : 3, accuracy, precision, recall, and *F*1-score were 0.930, 0.906, 0.991, and 0.946, respectively. The results show that the k-nearest neighbor model has better prediction performance when the data set is divided by 7 : 3.

## 4. Comparison and Analysis

In order to better understand the performance of the model established in this paper, this paper is based on the Python 3.9.7 development environment and uses the breast cancer data provided by Dr. William of the University of Wisconsin Clinical Medical Research Institute for experiments.

The experimental environment is Windows 11 operating system, the processor is Intel(R) Core(TM) i5-1155G7@2.50 GHz 2.50 GHz, and the memory is 8.00 GB.

The experimental parameters of each model are shown in Table 5, and the following three comparative analysis results will be carried out: (1) performance comparison of each model in this paper when the data set is divided into training set and test set in 8 : 2. (2) Performance comparison of each model in this paper when the data set is divided into a training set and test set in 7 : 3. (3) Comparison with some models in the literature [11–14].

1. When the data set is divided into a training set and test set by 8 : 2, the performance of each model is shown in Table 6.
2. As can be seen from Table 4, when dividing the training set and test set in a ratio of 8 : 2, the accuracy of the four machine learning methods is above 0.9, among which XGBoost and RF are above 0.95. The prediction accuracy of XGBoost is 0.974, indicating a high prediction accuracy. For precision, the precision of the KNN model is not high, below 0.9, only 0.888. XGBoost has the highest precision, which is 0.960. As for the recall, the recall of XGBoost, random forest, and logistic regression algorithms are all 1. K-nearest neighbor algorithm has the lowest recall, but it is also above 0.95. For this study, recall rates represent the proportion of malignant breast cancer samples that were correctly diagnosed. In medicine, it is very important for a disease to be diagnosed. The consequence of not being diagnosed is delayed treatment, which may result in patients missing the best time for treatment. This is much more serious than being diagnosed with a disease without having one. Therefore, the recall rate is a very important indicator in the field of disease diagnosis. Here, the recall of XGBoost, random forest, and logistic regression algorithm are all 1, indicating that all malignant breast cancers in the samples have been diagnosed. For *F*1-score, it can be seen that the *F*1-score of XGBoost, random forest, and logistic regression are all above 0.95. The *F*1-score of the XGBoost algorithm is the highest, reaching 0.980. The K-nearest neighbor model has the lowest *F*1-score of 0.934. Taking the four indicators into consideration, it can be said that when the data set is divided into a training set and test set by 8 : 2, the overall model effect of the XGBoost algorithm is better than the other three models. XGBoost not only achieved a recall of 1 but also achieved a recall of 0.95 or more for the other three metrics.
3. When the data set is divided into a training set and test set in 7 : 3, the performance of each model is shown in Table 7.
4. As can be seen from Table 7, when the training set and test set are divided by 7 : 3, the model effect of XGBoost and RF is obviously not as good as that of 8 : 2. First of all, in terms of the important index recall, when dividing the data set by 8 : 2, the recall of XGBoost and RF were both 1, but now they have decreased to 0.991 and 0.981, respectively. The two models also have slightly decreased in the other three indexes. However, the change of the prediction effect of logistic regression and the K-nearest neighbor model is different from these two algorithms. For the logistic regression model, the four indicators barely changed when the training set and test set were divided by 7 : 3 and 8 : 2, respectively. This shows that the logistic regression model is almost not affected by different data set partition. In the case of a 7 : 3 split between the training set and the test set, logistic regression showed lower accuracy, precision, and *F*1-score than XGBoost and random forest. However, the recall of the logistic regression model is 1, indicating that all malignant breast cancer has been predicted, which is of great significance for disease diagnosis. Therefore, it can be said that the prediction effect of the logistic regression model is better than the other three algorithms. For the KNN model, when the training set and test set were divided by 7 : 3, all four indexes increased. The recall increased to 0.991, which was higher than that of the random forest. The accuracy, precision, and *F*1-score increased from 0.912, 0.887, and 0.934 to 0.930, 0.906, and 0.946, respectively. Therefore, the KNN model performs better in the case of the training set and test set divided by 7 : 3 than the model divided by 8 : 2. The analysis shows that the division of data sets is not fixed. For different models, different divisions bring different changes in the model prediction effect.
5. According to the comparative analysis of Tables 6 and 7, the XGBoost model established in this paper (dividing the training set and test set by 8 : 2) has the best effect, and the following compares it with the model performance in the literature [11–14], and the specific results are shown in Table 8.

As can be seen from Table 8, the better performing models of the five models are the Logistic regression model of the literature [13] and the XGBoost model established in this paper. The recall and accuracy of the model in the literature [13] are 0.99 and 0.98, respectively, and the recall and accuracy of the model in this paper are 1.00 and 0.974, respectively, compared with the literature [13], the recall of the model is high, and the recall in medical diagnosis indicates the probability of detecting malignant cancer cells, which is of great significance for the classification of breast cancer cells, so the XGBoost model established in this paper has a better prediction effect and can be used as a medical tool to assist doctors to make treatment plans for breast cancer patients.

## 5. Conclusion

This paper mainly predicted the categories of breast cancer, from data preprocessing to feature selection, and then to the establishment of the model. Finally, the prediction results were compared and analyzed from many aspects.

In this paper, recall is taken as an important index to predict malignant breast cancer samples as accurately as possible. The original data set contained 30 features, and 15 features were selected as the input of the model through the Pearson correlation test. Before model construction, data were standardized to eliminate the impact of different dimensions on model effects. For the problem of unbalanced positive and negative samples, the stratified sampling method is used to extract training sets and test sets proportionally according to different categories of data. When selecting the optimal *k* value in the k-nearest neighbor, the recall is used as the model evaluation index, so that the *k* value with the highest recall rate is the optimal value.

The models are compared and analyzed from three aspects. The results are shown as follows:

1. In the case of dividing the training set and the test set by 8 : 2, the recall of XGBoost, random forest, and logistic regression is 1, which can predict all malignant breast cancer, and the K-nearest neighbor recall is slightly lower than 0.986 compared with the other three models. For the prediction accuracy, precision, and *F*1-score of the model, the results of the XGBoost model are better than the results of random forest and logistic regression, which are 0.974, 0.96, and 0.98, respectively, so the XGboost model is selected as the final prediction model under the condition of 8 : 2 division of the training set and the test set.
2. In the case of dividing the training set and the test set at 7 : 3, the values of the four evaluation indicators for XGBoost and random forest decreased, while the values of the four evaluation indicators for the K-nearest neighbor model were improved, but for the recall, only the recall of the logistic regression model was 1, and the other models were above 0.98, so the model prediction effect of logistic regression was the best, and the prediction accuracy, precision, and *F*1-score of logistic regression were 0.947, 0.922, and 0.96, respectively.
3. It can be seen from experiments that under different divisions, the prediction effect of the model has different changes. Comparing the optimal models in the two sets of different experiments, it can be seen that the prediction accuracy, precision, and *F*1-score of the XGBoost model (which divides the training set and the test set by 8 : 2) are higher than that of the logistic regression model (which divides the training set and the test set by 7 : 3) when the recall is 1, so the XGBoost model (which divides the training set and the test set by 8 : 2) works best in the model established in this paper. In addition, compared with the models in the literature [11–14], the XGBoost model established in this paper has a better effect and can accurately identify malignant breast cancer cells. However, this research is limited to numerical datasets, and in the future, we will try to use deep learning algorithms to apply various feature extraction techniques to image data (such as X-ray images) to obtain better classification results.

## Figures and Tables

**Table 1:** Data standardization results.

Radius_mean	Area_mean	Radius_se	⋯	Area_se	Radius_worst
1.0971	0.9844	2.4897	⋯	2.4876	1.8867
1.8298	1.9087	0.4993	⋯	0.7424	1.8059
1.5799	1.5589	1.2287	⋯	1.1813	1.5112
−0.7689	−0.7645	0.3264	⋯	−0.2883	−0.2815
1.7503	1.8262	1.2705	⋯	1.1904	1.2986

**Table 2:** Summary of filtering methods in feature selection.

Name	Variable type	Selection rules and variable requirements
Missing percentage	Univariate	Eliminate features that have too many missing samples and are difficult to fill
Variance		Exclude features with variance close to or equal to 0 apply to categorical variables
Frequency		Eliminate features that are overly concentrated on a certain category of values

Pearson's correlation coefficient	Multivariate	Features with correlation coefficients close to or equal to 0 are removed, but the sample needs to follow a normal distribution
Analysis of variance		Exclude features with an *F* value that is too low, or features with a *p* value <0.05. And the population sample is required to have homogeneity of variance and independence between samples
Kendall tau rank correlation coefficient		Exclude features with correlation coefficients close to or equal to 0, and require the categories to be ordered
Mutual information		Eliminate features with mutual information close to or equal to 0

**Table 3:** Characteristic variables and labels.

Number	Field	*ρ*_*X*_1_ *X*_2__
1	Radius_mean	0.730029
2	Perimeter_mean	0.742636
3	Area_mean	0.708984
4	Compactness_mean	0.596534
5	Concavity_mean	0.696360
6	Concave points_mean	0.776614
7	Radius_se	0.567134
8	Perimeter_se	0.556141
9	Area_se	0.548236
10	Radius_worst	0.776454
11	Perimeter_worst	0.782914
12	Area_worst	0.733825
13	Compactness_worst	0.590998
14	Concavity_worst	0.659610
15	Concave points_worst	0.793566
Label	Diagnosis (*M* = malignant, *B* = benign)	1.000000

**Table 4:** Confusion matrix.

Real situation	Predicted results	
	1	0
1	TP	FN
0	FP	TN

**Table 5:** Experimental parameters of each model.

Model	Parameter value
KNN	*n*_neighbors = 5
LR	Solver = “liblinear,” max_iter = 300
RF	Max_depth = 7
XGboost	Max_depth = 6

**Table 6:** The model effect of dividing the dataset by 8 : 2.

	Accuracy	Precision	Recall	*F*1-score
XGBoost	0.974	0.960	1.00	0.980
RF	0.965	0.947	1.00	0.973
LR	0.947	0.923	1.00	0.960
KNN	0.912	0.888	0.986	0.934

**Table 7:** The model effect of dividing the dataset by 7 : 3.

	Accuracy	Precision	Recall	F1-score
XGBoost	0.959	0.946	0.991	0.968
RF	0.953	0.946	0.981	0.963
LR	0.947	0.922	1.00	0.960
KNN	0.930	0.906	0.991	0.946

**Table 8:** Comparison of the results with some models in the literature.

Reference	Model	Recall	Accuracy
Literature [11]	XGBoost	0.958	0.979
Literature [12]	XGBoost	0.970	0.960
Literature [13]	Logistic regression	0.99	0.98
Literature [14]	Random forest	0.943	0.962
The model of this paper	XGBoost	1.00	0.974

## Data Availability

The data that support the findings of this study are openly available in UCI Machine Learning Repository at https://archive.ics.uci.edu/ml/datasets/Breast+Cancer+Wisconsin+%28Diagnostic%29.
